# The Impact of Socket Shield and Implant Surface Modification With Platelet-Derived Growth Factor on Osseointegration

**DOI:** 10.7759/cureus.69980

**Published:** 2024-09-23

**Authors:** Kshipra Kawadkar, Kaustubh Thakare, Simran Parwani, Priyanka Jaiswal, Aishwarya Rathod

**Affiliations:** 1 Periodontology, Vidarbha Youth Welfare Society Dental College and Hospital, Amravati, IND; 2 Periodontology, Sharad Pawar Dental College and Hospital, Wardha, IND

**Keywords:** growth factor, immediate dental implants, osseointegration, recombinant human platelet derived, socket shield

## Abstract

Implants have become the first treatment option in the anterior esthetic region. Buccal bone loss is the sequalae of tooth extraction compromising osseointegration and cosmetic goals. The present case report is of a 47-year-old female who requested to have her fractured central incisor replaced with fixed restoration supported by an implant; the tooth had already undergone a root canal and lacked a sufficient structure for crown prosthesis placement. This case report evaluates the efficacy of the socket shield technique (SST) combined with a coating of recombinant human platelet-derived growth factor-BB (rhPDGF-BB) on the implant surface to achieve optimal biomechanical stability and esthetics.

## Introduction

The principles of osseointegration have changed the therapeutic strategies of implant placement [1,2]. There is no doubt that initial stability while placing an implant plays a major role in acquiring osseointegration ensuring long-term esthetic success [3,4]. Besides this, esthetics are influenced by osseointegration, availability of bone, and surgical technique [5-7]. 

The socket shield technique (SST) is reliable as it helps preserve buccal bone, periodontal ligament fibers, and blood supply. Thus, reducing overall treatment duration and providing excellent esthetic results [8,9].

Recombinant human platelet-derived growth factor-BB (rhPDGF-BB) has been broadly investigated for its regenerative properties in the periodontal arena since the nineteenth century. Thus, to attain early osseointegration and gain better esthetics, SST adjunct to rhPDGF-BB as a surface implant modifier was employed.

## Case presentation

**Figure 1:**
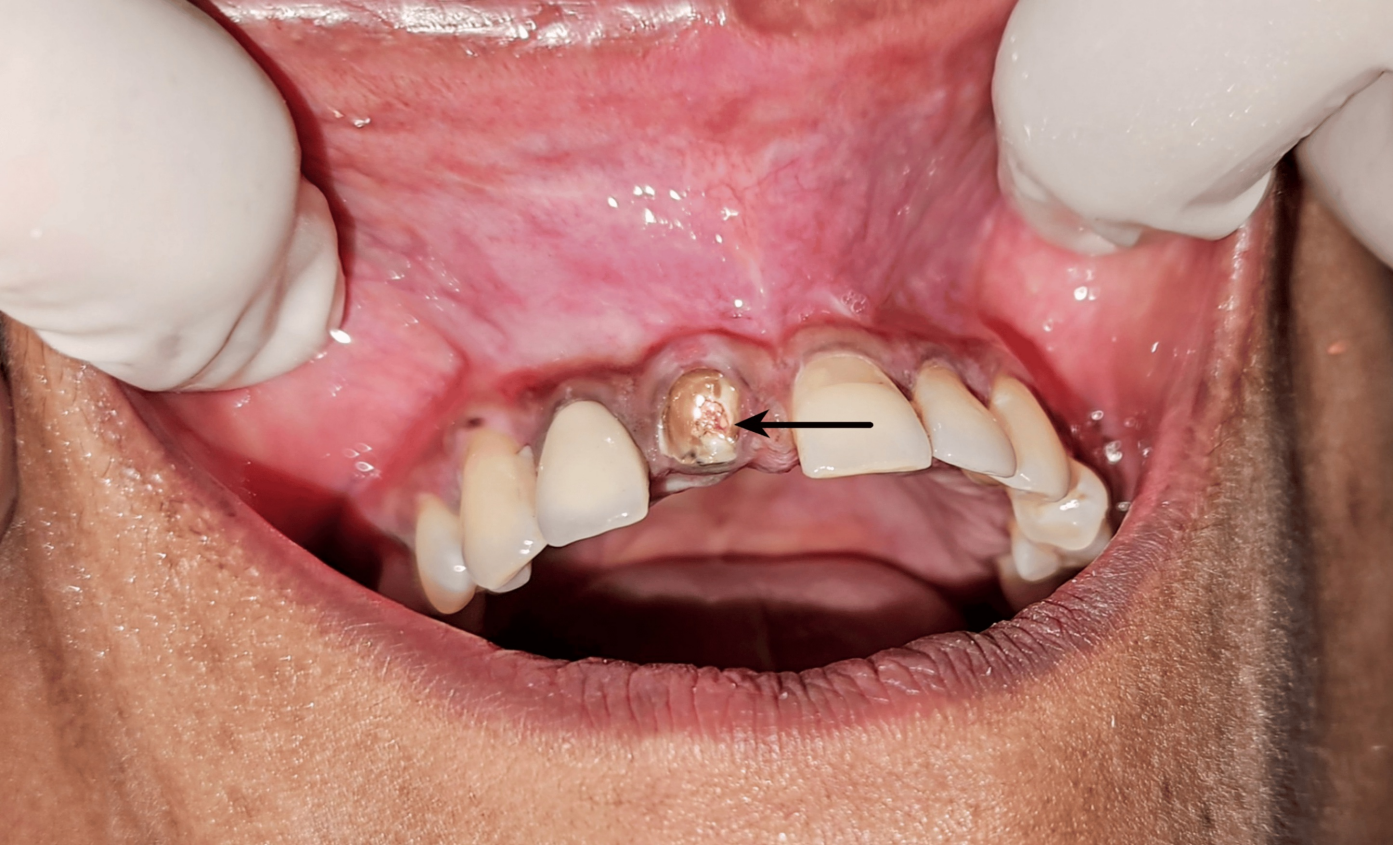
Endodontic failure with respect to tooth #11

A 47-year-old female patient reported a chief complaint of a fractured right central incisor to the outpatient department of periodontology. She was a discerning patient with specific superior esthetic requirements. On proper examination, it was observed that the right central incisor was lacking adequate keratinized mucosa and crown structure for achieving enhanced esthetics with immediate implant placement as evident in Figure 1.

**Figure 2:**
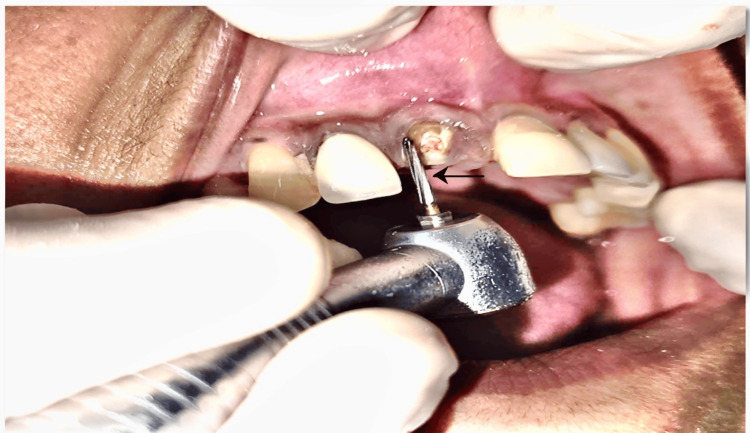
Horizontal sectioning with Zekrya bur

After performing phase I periodontal therapy, blood, and radiographic investigations, SST along with immediate implant placement was considered as treatment of choice. Initially, horizontal sectioning was carried out, followed by vertical sectioning with the help of 21mm Zekrya bur (carbide bur) as shown in Figure 2.

**Figure 3:**
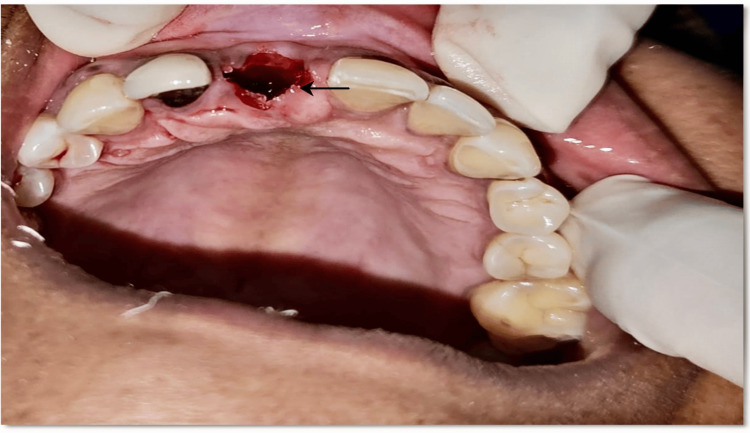
Buccal shield preparation

Later, gutta-percha was removed and a lingual root fragment was extracted. A buccal root fragment was retained which is observed in Figure 3, after beveling it to the level of alveolar crest.

**Figure 4:**
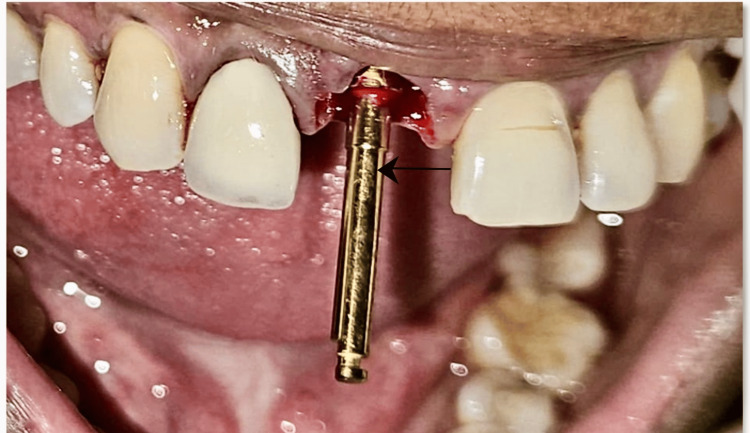
Osteotomy site preparation in labial view

Once the socket shield preparation was completed, the osteotomy site was planned as sighted in Figure 4.

**Figure 5:**
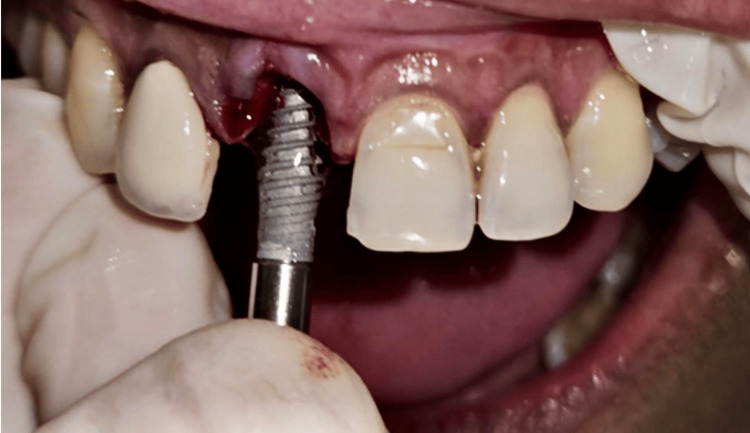
Following the placement path of osteotomy while implant placement

Parallelism was checked with adjacent teeth and osteotomy site preparation was accomplished. At this stage, 0.01% rhPDGF-BB gel commercially known as Plermin^TM^ was applied on the sterile implant surface with the aim of achieving early osseointegration. The implant was placed without damaging the buccal shield as observed in Figure 5. 

**Figure 6:**
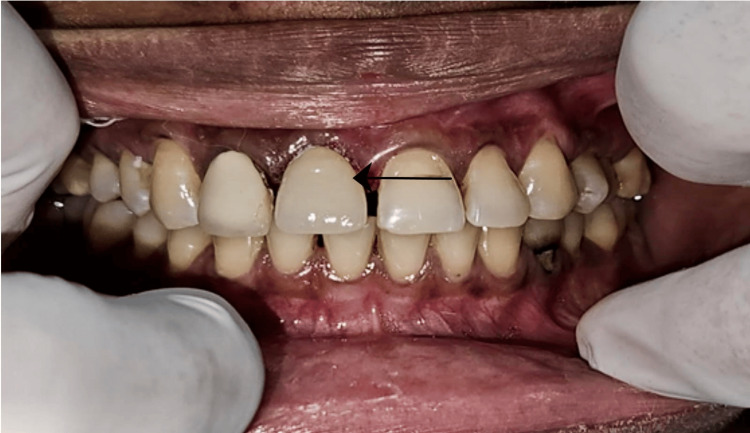
Prosthesis delivery after 3 months

Primary stability of 30N/Cm was achieved by a torque wrench. The use of SST made it possible to place an immediate implant without the need for bone grafting. Post-implant placement, a collagen plug was put down to prevent leakage of rhPDGF-BB gel and the site was sutured using a 3-0 polyglactin resorbable suture. An immediate post-operative radiograph revealed a prosthetically driven implant placement suitable for cement-retained prostheses. Prosthesis loading was done after achieving a good emergence similar to the adjacent tooth as seen in Figure 6.

The patient was advised to follow-up one, three, and six months post-operatively and exhibited excellent osseointegration and esthetic results with the help of a reverse torque test. 

## Discussion

The revolution in the field of implantology has led to the use of various implant surface bio-modifiers to increase biological stability. Recombinant human-derived PDGF-BB has been preferred in periodontal regeneration [10-12] but its use as an implant surface modifier has been only limited to animals. Similar findings were noted by Al-Hezaimi et al. and Kämmerer et al., who studied rhPDGF-BB to coat implants in beagle dogs and concluded that the use of rhPDGF-BB hastened the process of osseointegration together with an increase in bone to implant contact [13,14]. Though direct comparison of this case with other studies is not possible as previously no study had checked for rhPDGF-BB as an implant surface bio-modifier in humans. However, the findings of this case correspond to a study by Ghassib et al., who used rhPDGF-BB and collagen matrix in a sinus lift procedure and concluded that rhPDGF-BB was a successful treatment option to reduce overall treatment time as well as to achieve good osseointegration in the early healing phases [15].

With respect to the reverse torque test, the findings of this case match with Simeone et al., where the author found the reverse torque test to be a good prognostic tool to check the stability of 40 implants showing reduced incidence of failure in the first year of placement [16]. This case reported minimal gingival recession postoperatively, showing promising results of retaining the buccal shield. Similar to the present case, Dayakar et al. reported that the preservation of socket shield is a good option in esthetic regions [17].

Retaining shield and atraumatic partial extraction reduced attachment loss and contributed to superior esthetic outcomes. However, combining a socket shield with the topical application of rhPDGF-BB may yield confounding results and generate ongoing debate. Further clinical trials are required to put this technique into routine clinical practice.

## Conclusions

Results of this case report demonstrate successful implant placement in the maxillary central incisor in a female patient with high esthetic expectations. Thorough planning was done to achieve excellent esthetics and early osseointegration. The patient was satisfied with the functional and esthetic outcomes. Primary stability of 30N/cm was achieved which was greater than minimally required for predicting the success of the implant. Healing was uneventful and no post-operative complications were noted. SST in combination with the topical application of rhPDGF-BB on the implant surface helped preserve the buccal bone with negligible crestal bone loss. 

Advantages such as less minimal residual bone loss, preserving the keratinized mucosa, shorter treatment time and intervention, and excellent esthetics along with early osseointegration were emphasized by this case of SST along with rhPDGF-BB. Thus, SST in addition to rhPDGF-BB proved to be a boon in the treatment of maxillary anterior where immediate implant placement is otherwise complicated.
